# Protein signature in cerebrospinal fluid and serum of Alzheimer’s disease patients: The case of apolipoprotein A-1 proteoforms

**DOI:** 10.1371/journal.pone.0179280

**Published:** 2017-06-19

**Authors:** Chiara Fania, Beatrice Arosio, Daniele Capitanio, Enrica Torretta, Cristina Gussago, Evelyn Ferri, Daniela Mari, Cecilia Gelfi

**Affiliations:** 1 U.O. Proteomica Clinica, IRCCS Policlinico San Donato, San Donato Milanese (MI), Italy; 2 Geriatric Unit, Department of Medical Sciences and Community Health, University of Milan, Milan, Italy; 3 Fondazione Ca’ Granda, IRCCS Ospedale Maggiore Policlinico, Milan, Italy; 4 Department of Biomedical Sciences for Health, University of Milan, Segrate (MI), Italy; 5 Nutritional Sciences, University of Milan, Milan, Italy; Nathan S Kline Institute, UNITED STATES

## Abstract

In the diagnosis of Alzheimer’s disease (AD) total tau (T-tau), tau phosphorylated at threonine 181 (P-tau181), and the 42 amino acid isoform of alpha β-amyloid (Aβ) are well established surrogate CSF markers. However, there is a constant need for new diagnostic markers to identify the disease at a very early stage. The identification of new molecules for AD diagnosis and monitoring in CSF is hampered by several “confounding” factors including intra- and inter-individual, pre-analytical and analytical variabilities. In an attempt to partially overcome patient’s variability and to determine new molecules significantly dysregulated in CSF, we assessed the proteome profile of low molecular weight protein species in CSF and serum of the same patients. CSFs and sera from 36 ADs, 32 iNPHs (idiopathic normal pressure hydrocephalus) and 12 controls were compared by MALDI profiling (non-parametric statistics, CV<20%, AUC>0.750). After protein identification by mass spectrometry, the proteoform composition was assessed by 2-D DIGE/MS. Results indicated that CSF of iNPH can be used as control. Serum and CSF of AD patients shows a specific protein profile compared to iNPH samples. A variation (p<0.01) of Apo A-1 levels in AD, together with a specific dysregulation of Apo A-1 proteoforms was observed. The profiling of CSF and serum of the same patients, suggests that the decrement of total Apo A-1 occurs specifically in CSF. Serum and CSF of AD shows a characteristic Apo A-1 proteoform pattern suggesting it as potential marker which can support the clinical workflow adopted for AD diagnosis and progression.

## Introduction

Dementia is one of the biggest global public health challenges facing our generation. Recent data estimates that 66 million people will be affected by 2030 and 115 million by 2050 worldwide [1]. Alzheimer's disease (AD) is the most common form of dementia and its differential diagnosis includes normal ageing, other dementias, symptomatic confusional states and mood disorders [2, 3]. Current protocol for AD diagnosis includes: magnetic resonance imaging (MRI) to assess brain atrophy, amyloid beta (Aβ) pathological load using positron emission tomography, assessment of tau and Aβ in cerebrospinal fluid (CSF) and APOE polymorphisms. The major issue of the assessment of the above mentioned molecular biomarkers is represented by the variability of results, making it difficult, in some cases, to provide a proper diagnosis and predict the evolution of mild cognitive impairment to AD. Total tau (T-tau), tau phosphorylated at threonine 181 (P-tau181), and the 42 aminoacid isoform of alpha β-amyloid (Aβ42) dysregulated levels are a characteristic of AD patients and are used to support clinical diagnosis. Nevertheless, their concentration depends on several “confounding” factors related to pre-analytical variables like sample collection modality, material of diagnostic tubes, CSF handling and storage. Recently, specific rules on CSF withdrawal and treatment have been defined to standardize methodologies including type of tubes, centrifugation, transfer and storage [4]. Furthermore, a novel assay including the assessment of the ratio of amyloid Aβ 40/42 ratio indicates a better performance of the test compared to Aβ42 alone [5]. Moreover pre-analytical and analytical variables have been properly addressed by recent studies, the intra- and inter- individual variability still remains a critical point. Beside the principal aim of identifying new candidates for AD diagnosis and monitoring, this study deals with the intra and inter-individual variability and the low sample number availability. This was addressed by performing the proteome profiling of low molecular weight proteins for CSF and low abundant low molecular weight proteins for serum to detect discrepancies of specific proteoforms [6] between blood and CSF in AD patients compared to controls. Patient clusterization was based on tau, p-tau, and Aβ levels.

Many research groups sought the detection of signals directly in the blood by proteomic analysis, adopting either a gel-based approach [7] or direct analysis of pre-labelled trypsin digested samples by LC-MS [8]. These proteomic studies were mainly performed either on serum or CSF, whereas fewer studies have highlighted proteome composition and differences between CSF and serum of the same subject. This could be due to the limited availability of CSF in healthy controls. All these proteomic studies were mainly case-control and, more importantly, the protein recognition by MS was made on enzyme digested protein samples following a “bottom-up” proteomic strategy [7, 9].

Unfortunately, all information about the structure and the nature of proteoforms, resulting from PTMs (post-translational modifications) or protein truncation, are lost due to the step of the protein digestion, adopted prior to protein identification [10]. Furthermore, PTMs can be analyzed in a targeted fashion only.

The need of a multiplexed panel of proteins and putative biomarkers involved in AD and neurodegeneration, is demonstrated by a recent paper in which biomarkers detected in plasma have been selected as indirect indicators of AD pathology and their value assessed as predictors for future disease conversion [11].

Although sensitivity and specificity of this panel of biomarkers were quite good, some results appear contradictory, as they analyzed data from different studies. This suggests the need for a more sharpened proteomic study based on intact proteins differentially present in CSF and serum of the same patient. This approach can facilitate the validation step.

Particular interest has been recently addressed to apolipoprotein A-I (Apo A-1) the major constituent of human high-density lipoproteins, which plays a key role in reverse cholesterol transport and lipid homeostasis [12]. Apo A-1 exhibits antioxidant and anti-inflammatory properties [13] and inhibits the aggregation and neurotoxicity of amyloid-β peptide in AD [14]. Although the possible association between apolipoproteins and neurodegeneration is unclear, increased Apo A-1 levels were correlated with decreasing risk of dementia [15], raising the possibility of a novel role of Apo A-1 in protection against neurological disorders [16–18]. However, recent results on Apo A-1 levels and their correlation with dementia appear contradictory. In AD, Apo A-1 levels were described as decreased or unchanged [7, 19], whereas hyper-methylation of the promoter region and the concurrent switch-off of the APOA1 gene, recently documented [19], would suggest a decrease of this protein in AD. However hyper-methylation was not supported by ELISA results, leaving the question of Apo A-1 abundance still open.

## Materials and methods

### Patients

This study involved 36-AD, 32-iNPH, and 12-healthy age-matched subjects (Table 1 and S1 Table), enrolled by Fondazione Ca’ Granda, IRCCS Ospedale Maggiore Policlinico, Milan, Italy.

**Table 1:** Participants demography.

	AD	iNPH	Controls
**Patients, N (males—females)**	36 (12–24)	32 (19–13)	12 (9–3)
**Median Age, years (min-max)**	77 (68–86)	83.5 (70–91)	75.5 (55–84)
**Median CSF Aβ, pg/mL (min-max)**	467 (226–1151)	747 (250–1570)	1062 (453–1515)
**Median CSF Tau, pg/mL (min-max)**	573.5 (110–2952)	159 (46–676)	95 (49–149)
**Median CSF p-Tau, pg/mL (min-max)**	72 (18–475)	29 (15–73)	29 (7–37)

All participants gave their informed consent to the study, including medical history, physical and neurological examination, neurocognitive evaluation (Mini-Mental State Examination), computed tomography or MRI scan, and screening laboratory tests consisting in the assessment of levels of tau, phospho-tau (p-tau), and amyloid-β (Aβ) proteins by ELISA (Innogenetics). AD patients fulfilled the NINCDS-ADRDA criteria [20] and those proposed by McKhann et al. [21]. The iNPH subjects were diagnosed according to International Guidelines published in 2005 [22]. Control subjects were likewise examined to exclude the presence of neurological and cognitive disorders.

CSF samples were drawn in polypropylene tubes after lumbar puncture at the L4/L5 or L3/L4 interspace, centrifuged at 4°C and stored at ≤ −80°C until analysis.

Blood samples were collected in vacutainer tubes and placed at 4°C for 15 min until clotted. Samples were centrifuged for 30 min at 3000 rpm at 4°C and sera transferred into 1.8 mL cryovials (Thermo Fisher Scientific) and stored at −80°C.

### MALDI profiling

Protein concentration was determined by BCA assay (Pierce). One ug of each CSF sample of AD, iNPH and healthy subjects were diluted 1:1 (w/V) in DHAP (2,5-dihydroxyacetophenone) matrix (15 mg/mL in 3:1 ethanol:diammonium hydrogen citrate, 5% trifluoroacetic acid), analyzed in four replicates by MALDI profiling using an AnchorChip plate (800–384 target, Bruker Daltonics), and dried at room temperature. For serum analysis, samples were immunodepleted from the 6 most abundant proteins and subjected to mass spectrometric analysis as previously described [23, 24].

Spectra from 20-AD patients, 20-iNPH cognitive-healthy subjects and 12-healthy subjects (from here named “controls”) were acquired in linear positive modality (low resolution modality) using an Ultraflex III mass spectrometer equipped with Smartbeam laser, frequency of 100 Hz, FlexControl software v. 3.3, and FlexAnalysis software v.3.3 (Bruker Daltonics). Spectrometer and spectra acquisition settings were as described in [23]. Spectra were analyzed by ClinProTools software v. 2.2 (Bruker Daltonics) using 800 resolution, Top Hat Baseline, 10% minimal baseline width, Savitsky-Golay smoothing as spectra parameters. Statistics was performed by ClinProTools software, a post-processing tool which automatically manages raw spectra (baseline subtraction, spectra recalibration, and peak normalization) in order to obtain data suitable to perform statistics.

### MS data statistics

Spectra from AD and iNPH patients and controls underwent cluster and univariate analysis of individual peaks (CV≤20%) by ClinProTools software. In particular, the Wilcoxon/Kruskall-Wallis’ t-tests (significance level for p-value<0.05) were adopted, and generated a list of signals differentially expressed in AD, iNPH and controls and those with p<0.05 were named “best separating” peaks.

The discriminatory power of each best separating peak was further assessed using the receiver operating characteristic (ROC) area-under-the-curve analysis (AUC>0.750) whereas peaks’ distribution was determined by box plots.

When needed, effect size evaluation was performed using the standardized effect size “d” by Cohen.

To verify if the best separating peaks discriminate different types of dementia from controls, a Quick Classifier algorithm was adopted and tested using an independent set of samples in blind (validation set) of 36 samples (24-AD and 12-iNPH) to determine the sensitivity and specificity of the proposed analysis.

The Quick Classifier is a univariate algorithm and is a key feature of ClinProTools software, allowing to sort the class average of peak areas together with statistical parameters (such as p-value) for each selected peak.

### Peak identification by SDS-PAGE and MALDI-ToF mass spectrometry

SDS electrophoresis was performed on serum protein extract pools of 9-ADs, 9-iNPHs and 9-healthy subjects (60 ug per sample, 4% stacking gel pH 6.8, 20–27% T gradient for running gel pH 8.8). Gels were stained with SYPRO Ruby (Molecular Probes) and acquired by Ettan DIGE Imager (EDI, GE-Healthcare). Molecular weight markers (Sigma-Aldrich) were run in a separate lane.

After separation, protein bands were excised, and analysed by using an Ultraflex III mass spectrometer as described in [25]. Proteins were identified by comparing experimental peaks with a database of in silico tryptic peptides from known proteins using in house MASCOT software v.2.2. Search was carried out by correlation of uninterpreted spectra to Homo sapiens entries in NCBInr database (11833178 sequences; 4040378175 residues). One missed cleavage per peptide was allowed and carbamidomethylation was set as fixed modification while methionine oxidation as variable modification. Peptide mass tolerance was set at 30 ppm.

### Immunoblotting

Fifty ug of proteins from crude sera and CSFs from 4-AD, 5-iNPH patients and 4-controls, respectively, were separated by SDS-PAGE (14% T, pH 8.8 running gel), transferred and blocked onto a PVDF membrane (300 mA; 180 min) utilizing a Transblot Cell (GE-Healthcare). The membranes were incubated with monoclonal anti-Apo A-1 (1:400 as primary antibody; Santa Cruz Biotechnology, Inc.) and HRP conjugate anti-mouse (1:4000 as secondary antibody; GE-Healthcare). Proteins were visualized by chemiluminescence using ECL Plus kit (GE-Healthcare).

### Two-dimensional difference in gel electrophoresis (2-D DIGE)

Protein labeling, 2D-separation, and semi-quantitative analysis were performed as previously described [25]. Specifically, the sample proteins from serum and CSF of AD, iNPH and healthy controls, respectively, were labeled with Cy5 whereas the internal standard was labeled with Cy3. Samples from serum of 20-AD, 20-iNPH patients and 12-controls were sub-pooled by combining 4 different samples randomly selected within AD, iNPH and control. This approach is justified when few samples are available and the variance is high. In this case, it is expected that the experiment will have a low power (i.e. the ability to detect changes in expression) and hence, will be ineffective in detecting expression changes. The method of sub-pooling enables to reduce the variance among biological groups increasing the power to detect changes in expression above the average value of sub-pooled sample [26, 27]. This approach allowed to obtain five biological replicates (composed by 4-samples for each pool) for AD and iNPH, and 3 biological replicates (composed by 4-samples for each pool) for controls were made both for serum and CSF. Pooled samples (40 ug/ pool) were combined with an equal amount of internal standard. Each pooled sample was run on 18 cm, 4−7 linear pH-gradient IPG strips, second dimension was performed as described in [25].

CyDye-labeled gels were acquired using an EDI imager. Spot detection was performed using DeCyder DIA module V. 6.5 (difference in-gel analysis, GE-Healthcare) while inter-gel matching and statistical analysis were obtained adopting DeCyder BVA module V. 6.5 (biological variance analysis, GE-Healthcare). Statistically significant differences were computed by One-way ANOVA and Tukey tests (significance level was set at p<0.01). False discovery rate was applied, as multiple test correction, in order to keep the overall error rate as lower as possible. Only proteins with spot volumes consistently different in all replicates, were considered differentially expressed.

### Spots identification by MALDI-ToF MS

Semi-preparative gels, containing 400 ug of total protein extract per strip, were loaded; electrophoretic conditions were the same as for 2-D DIGE, except that gels were stained with a protein fluorescent stain (Deep Purple Total Protein Stain, GE-Healthcare). Image acquisition was performed using the EDI imager. Protein identification was carried out as described previously, and MS data are reported in Supporting Information.

## Results

### CSF MALDI profiling for control selection

For comparative studies the choice of the proper reference is crucial. In our case the control should be a subject with CSF available, without symptoms of dementia and negative to AD markers. Idiopathic normal-pressure hydrocephalus (iNPH) were selected as possible reference. These patients are characterized by reversible symptoms of dementia (gait impairment, cognitive decline and urinary incontinence) related to an increased production of CSF leading to a pressure overload causing transient symptoms [28]. However, their diagnosis is routinely performed by using the assessment of physical symptoms, coupled to neuroimaging approaches and CSF tap-test [20].

**Fig 1:**
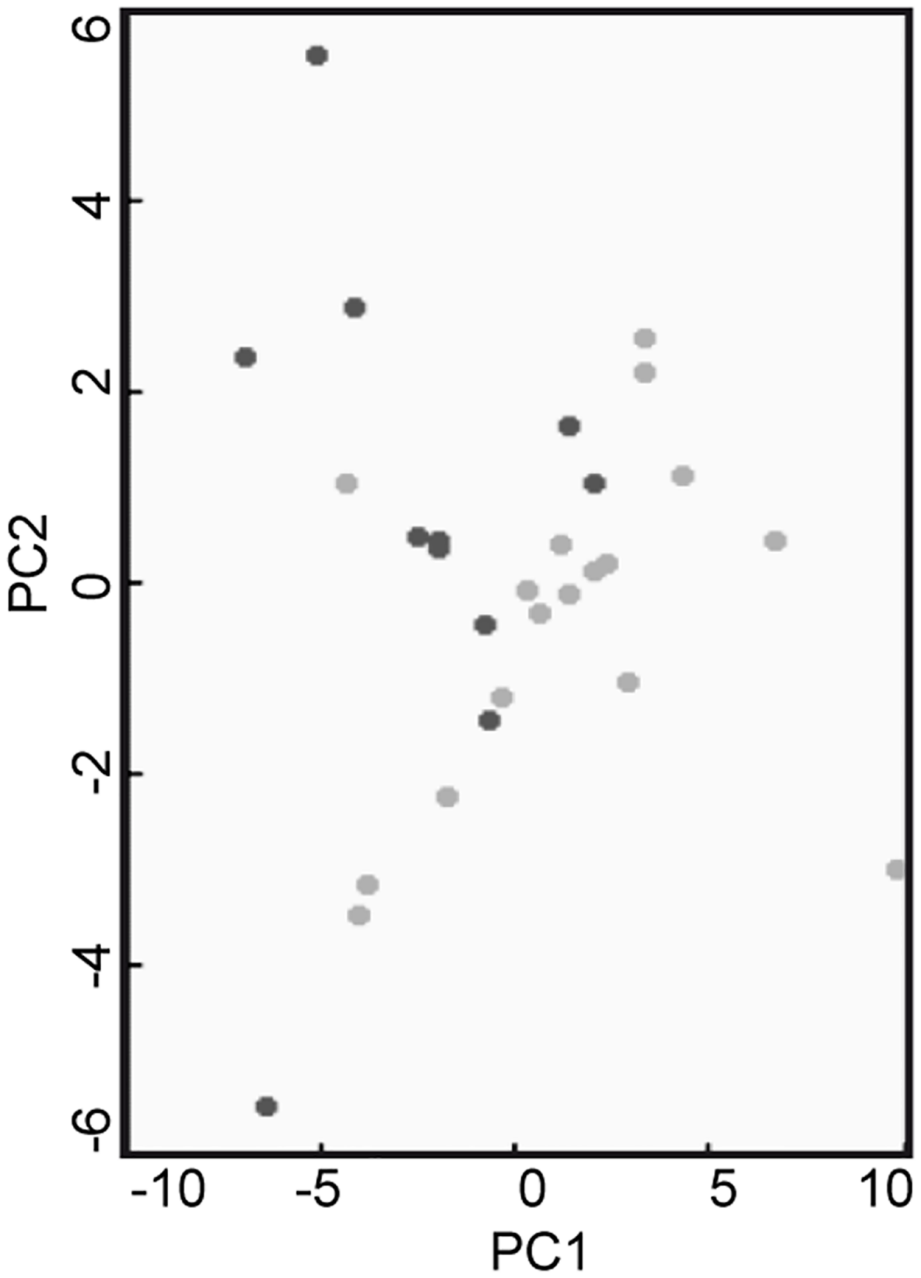
PCA score plot relating to healthy controls *vs* iNPHs comparison. PCA plot is based on the CSF profiling of healthy subjects (black circles) and iNPH patients (grey circles). The analysis, performed by using ClinProTools software showed the absence of clusterization of the two classes.

However, before utilizing them as reference, a comparison with control subjects neurologically healthy was performed by MALDI profiling. Spectra of CSF from iNPH (n = 20) and cognitively healthy subjects (n = 12) were acquired by MALDI-MS and compared utilizing the ClinProTools software statistic module. No differentially expressed peaks in the m/z range 4000–35000, typical of MALDI profiling, were revealed, as shown by the PCA plot of Fig 1, indicating that, in this m/z range, the protein composition was comparable and CSF from iNPH patients can be used as reference group, overcoming the issue of the recruitment CSF from control samples.

### CSF MALDI profiling of selected cohorts

**Fig 2:**
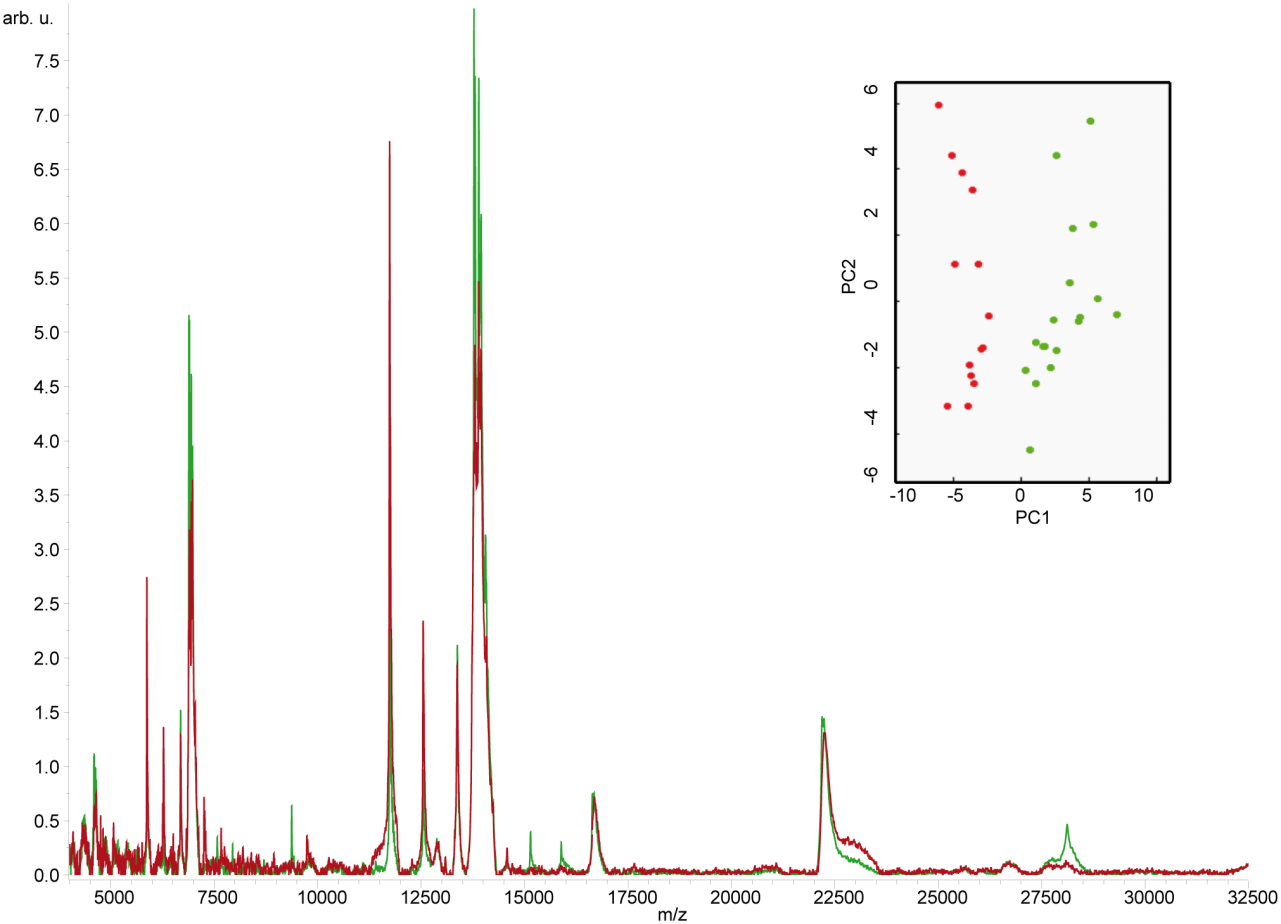
CSF MALDI profiling of AD *vs* iNPHs. Representative MALDI profiling spectrum of CSF from AD (average spectrum, red line) and iNPH patients (average spectrum, green line). Samples were spotted onto the AnchorChip target (Bruker Daltonics) using DHAP matrix as described in method section. The corresponding PCA plot showed a good separation between the considered classes (AD, red circles; iNPH, green circles).

**Fig 3:**
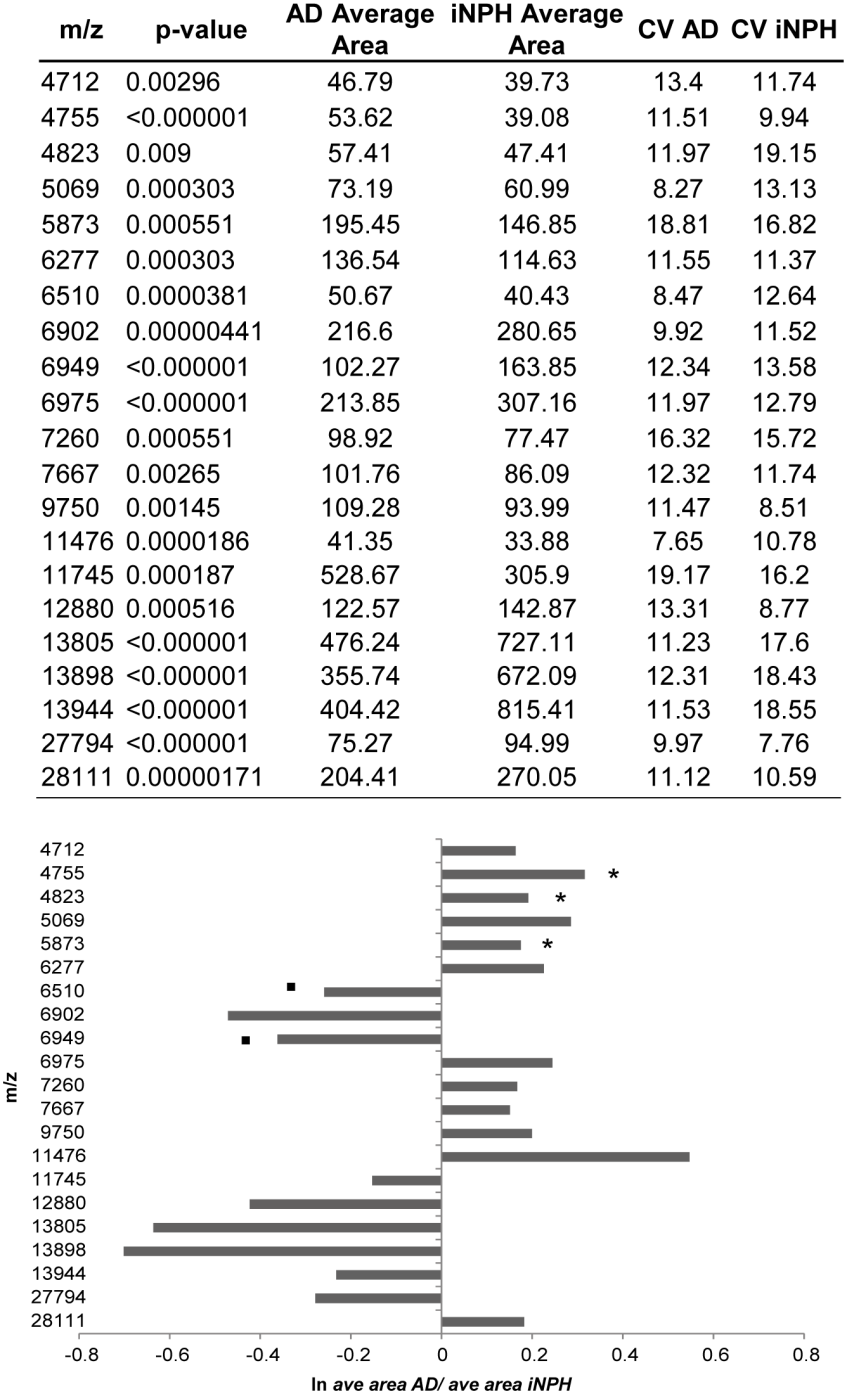
Dysregulated peaks in AD vs iNPH comparison. Upper panel: differentially expressed peaks (p<0.05) in CSF of AD vs iNPH patients. For each peak, p-value obtained adopting a non-parametric test (Wilcoxon test), class average areas and CVs are indicated. Lower panel: changed peaks expression in CSF reported as ln of the ratio of the average area for each class. Asterisks and dots indicate dysregulated peaks both in CSF (see paragraph “CSF MALDI profiling of selected cohorts”) and in serum (see paragraph “Protein levels of total Apo A-1 in serum”) with same/opposite trend, respectively.

CSF from AD (n = 20) and iNPH (n = 20) were analyzed utilizing the same MALDI profiling procedure. The analyses identified a protein pattern characteristic of AD, as indicated by the PCA plot with 21 peaks differentially expressed (p<0.01, CV<20%) in the m/z range 4000–35000 (Figs 2 and 3).

Among them, 9 were under-expressed whereas 12 were over expressed in AD compared to iNPH control samples, indicating that a protein signature of CSF of AD patients is detectable by MALDI profiling analysis.

Aware of the limited number of samples, the standardized effect size “d” was evaluated for dysregulated peaks, indicating a consistent difference in the areas, on average, between ADs and iNPHs.

### Generation of predictive models

**Fig 4:**
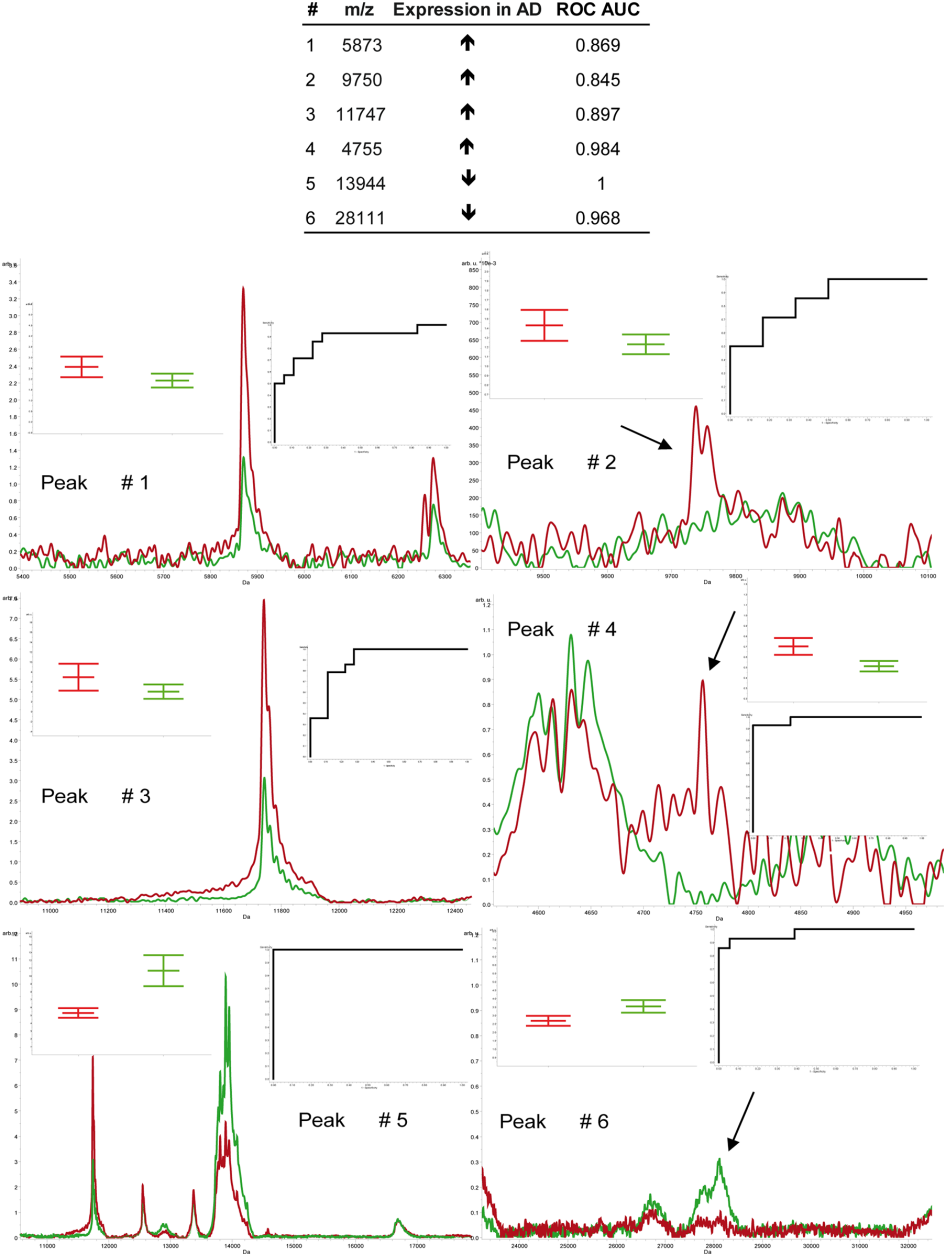
Peaks with more stringent statistics from the comparison AD (average spectrum, red line) vs. iNPH patients (average spectrum, green line). In the upper region, the peaks are listed together with their expression trend in AD and ROC AUC. In the lower region, the close-ups of selected peaks are shown together with box plots and ROC curves. Spectra were acquired by MALDI-MS in linear positive modality and statistics was performed by ClinProTools software.

The selection of putative proteoform biomarkers was carried out using statistics. Twenty-eight samples underwent MALDI profiling analysis as described below in a blinded fashion. The classification model was generated selecting peaks characterized by non-overlapping box plots, Wilcoxon p<0.01, CV<20% and AUC>0.800. This approach revealed 6 best candidate peaks (Fig 4).

By using the Quick Classifier algorithm, provided by the ClinProTools software, these peaks were tested alone and in combination to find the best model for classifying unknown CSF samples. Values of sensitivity and specificity obtained for each model are shown in Table 2.

**Table 2:** Peaks combination for the generation of models for blind samples. Models were constructed by Quick Classifier module of ClinProTools software by combining the peaks with the most robust statistics. For each combination, the name of the model, sensitivity, specificity and false negatives are shown.

Model	Peaks Used in Model Construction	Sensitivity (%)	Specificity (%)	False Negatives (%)
A	1	85	82	2.8
B	2	86	83	5.6
C	3	85	83	8.3
D	4	82	75	11.1
E	5	69	17	13.9
F	6	95	92	16.7
G	1, 6	95	92	11.1
H	1, 5	71	33	8.3
I	2, 6	94	92	22.2
J	2, 5	69	25	11.1
K	3, 5	75	42	8.3
L	3, 6	95	92	16.7
M	4, 6	89	83	22.2
N	4, 5	76	50	11.1
O	1, 4, 5, 6	85	75	19.4
P	1, 2, 5, 6	78	58	16.7
Q	1, 3, 5, 6	89	83	19.4
R	2, 3, 5, 6	79	58	13.9
S	1, 2, 3, 4, 5, 6	84	75	22.2

Best results were obtained adopting F, G and L models whose sensitivity (i.e. the proportion of predicted AD cases who actually have AD), and specificity (i.e. the number of predicted controls who are actually controls) are 95% and 92%, respectively. Although these three models showed the same performance, model G was characterized by a lower number of false negatives (11.1% of false negatives) compared to F and L models (both 16.7% of false negatives), suggesting G model as best classifier for unknown samples. Importantly, a peak with m/z of 28000, less abundant in AD compared to iNPH (see S2 Table for area values utilized by ClinProTools software for statistics), was shared by F, G and L models, indicating the need to identify this signal.

### Peak identification

CSF protein samples from AD and iNPH patients were separated by SDS-PAGE. After protein separation, the band at 28000 Da was gel-excised and analyzed by MALDI-MS. MS analysis identified apolipoprotein A-1 (MASCOT score 121, 11 identified peptides, 34.5% of sequence coverage, S3 Table and S1 Fig), which was less expressed in AD respect to iNPH (~24%) by MALDI profiling of CSF.

### Protein levels of total Apo A-1 in serum

To clarify whether Apo A-1 levels were comparable in CSF and serum collected from the same patient, the MALDI profiling approach was adopted to assess the profile of intermediate abundant proteins (from mg to ug). Sera of AD and iNPH patients, and healthy controls, already enrolled for CSF analysis, were immunodepleted by Hu7 column as described in methods and eluted proteins, profiled by MALDI-MS. Overall, 37 peaks were differentially expressed (p<0.05). Among them, a number of signals with the same m/z were present in both fluids and, in some cases, they show an opposite trend (Fig 3, lower panel). It should be specified that the nature of these peaks (currently under identification in our lab) is out of the aim of the present paper.

**Fig 5:**
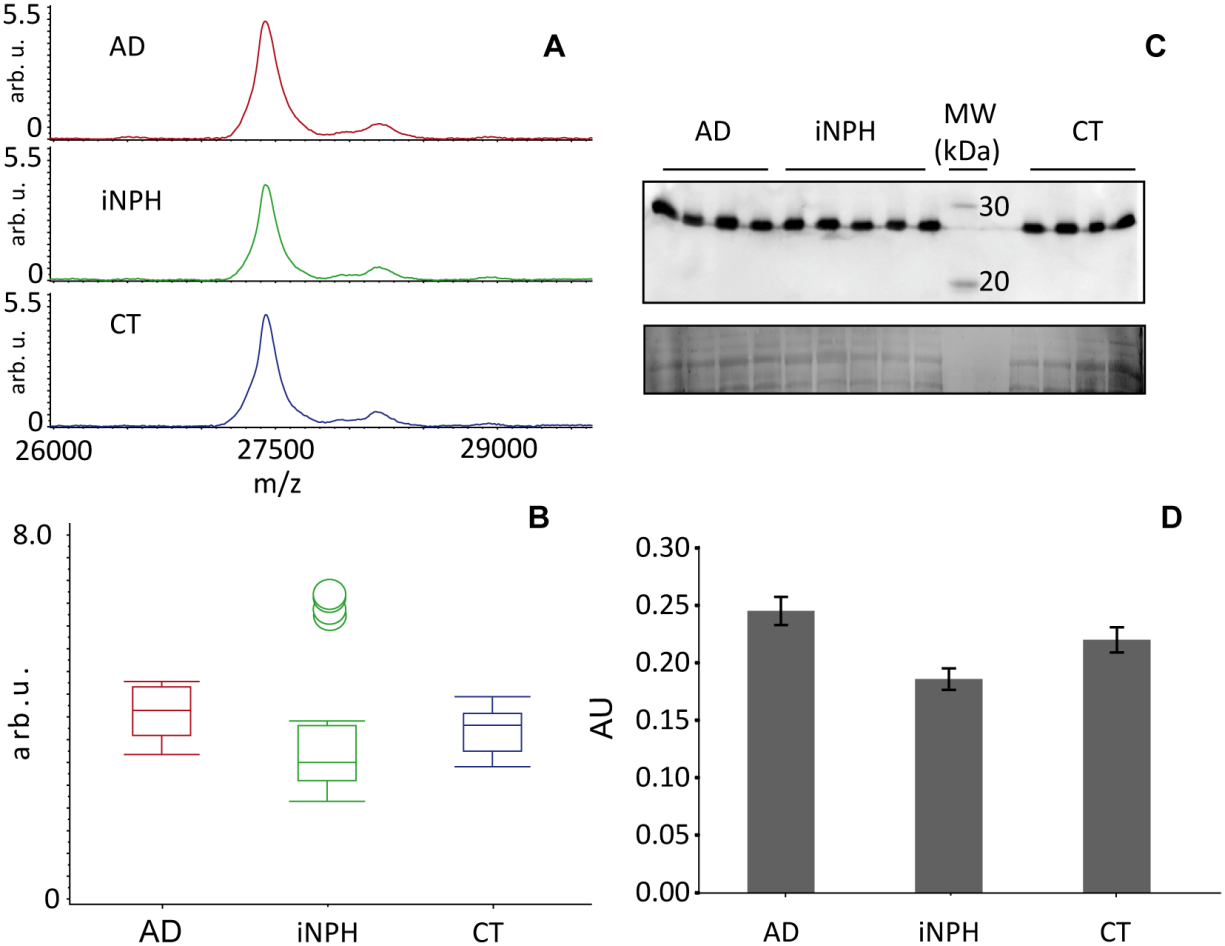
Total Apo A-1 expression analysis. **A)** Apo A-1 peak obtained after MALDI profiling of Hu7 immunodepleted serum (m/z range 1500–35000) from AD (average spectrum, red line), iNPH patients (average spectrum, green line), and controls (average spectrum, blue line), showed **B)** an unchanged expression among classes. **C)** 50 ug of crude sera from AD and iNPH patients and controls were separated in a 14% T, 4% C polyacrylamide gel, pH 8.8, after separations proteins were transferred to PVDF membranes and incubated with anti-Apo A-1. Normalization was performed from the Sypro Ruby total protein staining. The analysis confirmed the unaltered total Apo A-1 expression as shown by the corresponding box plots **(D)**.

Conversely to CSF analysis, the peak corresponding to Apo A-1 was unchanged (Fig 5A and 5B and S4 Table) even if a slight, non-statistically significant increment in AD, was observed. The total Apo A-1 expression was further validated by blotting, which confirmed the unchanged levels in AD patients (Fig 5C and 5D).

### Apolipoprotein A-1 proteoforms’ pattern characterization

With the aim to investigate Apo A-1 as a potential diagnostic marker, the quantification of the relative abundance of each specific proteoform compared to total protein assessment was pursued.

**Fig 6:**
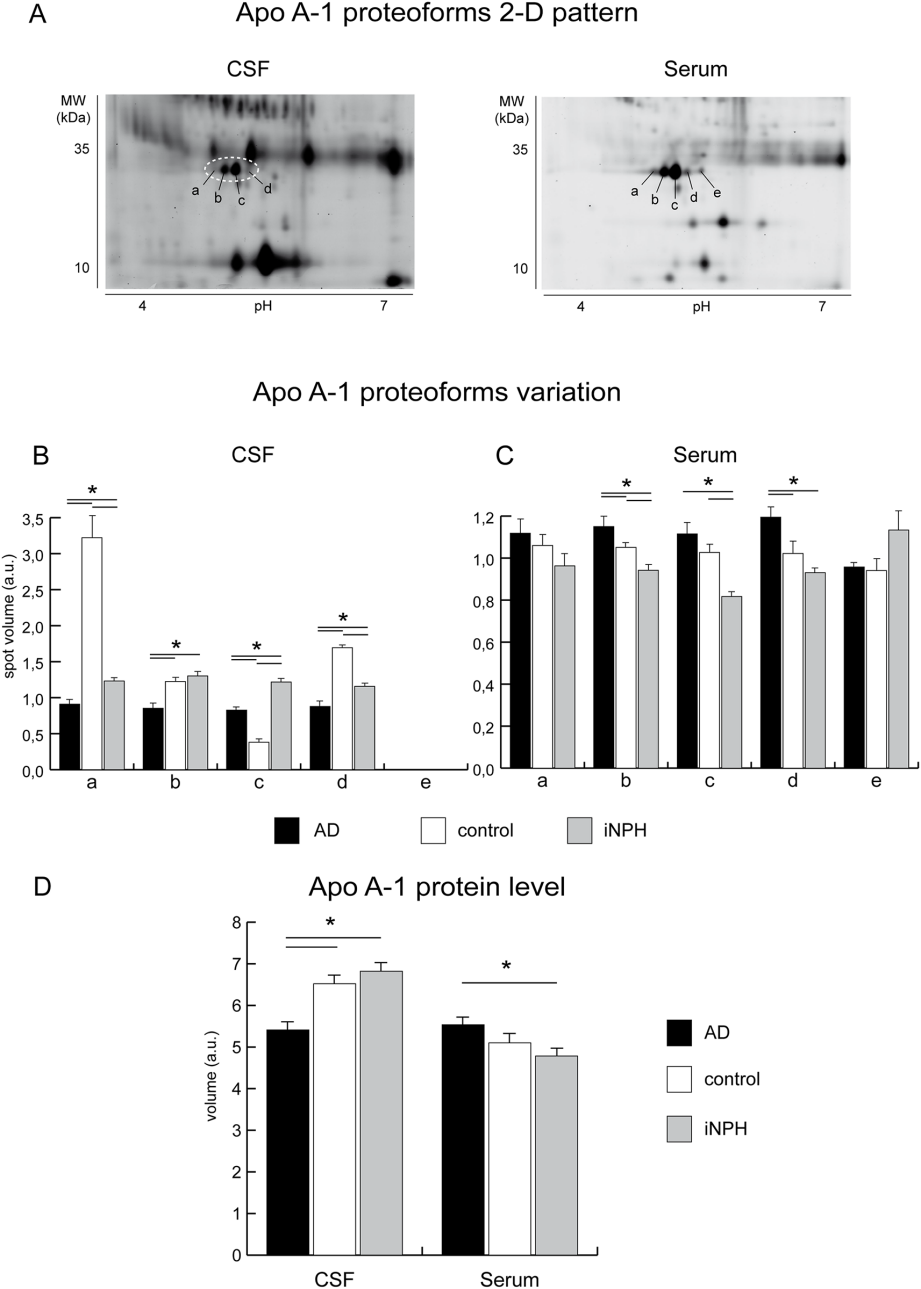
Apo A-1 expression analysis. **A)** Representative close-up of 2-D DIGE map of CSF and serum protein extracts. Protein extracts (40 ug) were separated in a pH 4–7 IPG strip (first dimension) and in a 12% T, 2.5% C polyacrylamide gel (second dimension). Letters a, b, and c indicate the Apo A-1 proteoforms at pI 5.13, 5.20, 5.27, respectively, whereas d and e represent the proApo A-1 proteoforms at pI 5.35 and 5.42. **B)** Differential analysis of AD (black bars) CSF compared to controls, (white bars) and iNPH (grey bars) (statistical analysis performed using DeCyder software, BVA module) revealed that Apo A-1 proteoforms a, b, c and d (panel A, dotted circle) were at variance (One-way ANOVA and Tukey tests, p-value<0.01) in AD and iNPH versus control samples. Specifically, the proteoform a, was decreased in AD compared to controls, the proteoform b, was unchanged in iNPH and controls whereas was decreased in AD compared both to iNPH and controls. The proteoform c increased in AD versus controls, whereas it was decreased compared to iNPH. Finally, proteoform d resulted decreased in AD compared both to iNPH and controls. Proteoform e remained undetected in CSF. Collectively these results suggest that Apo A-1 downregulation compared to controls and iNPH, targets specific proteoforms. **C)** Differential analysis of AD (black bars) sera compared to controls (white bars) and iNPHs (grey bars) also revealed a complex pattern of dysregulation. The proApo A-1 proteoform e, was unchanged, whereas the proteoform d was increased in AD compared to iNPH and controls. The mature Apo A-1 proteoform c was increased in AD and decreased in iNPH compared to controls, the b proteoform increased in AD and decreased in iNPH compared to controls whereas a was unchanged (One-way ANOVA and Tukey tests, p-value<0.01). (**D**) Differential analysis of total Apo A-1 in CSF and serum of AD (black bars), cognitively healthy controls (white bars) and iNPHs (grey bars). Asterisks = t-test p<0.05. Concerning the total amount of APOA-1 by DIGE, which results from the sum of volumes of proteoforms, it was significantly downregulated in CSF in AD *versus* iNPH. In serum the total proteoforms volume indicates an overall increment of Apo A-1 in AD compared both to controls and, with a statistical significance, to iNPH, supporting a trend observed by MALDI profiling and by SDS blotting.

The expression pattern of Apo A-1 proteoforms in CSF and in serum of AD, iNPH patients, and controls, was characterized by 2-D DIGE (S5 and S6 Tables). This technology enables the separation of intact proteins based on their isoelectric point, in the first dimension, combined with the apparent MW assessment, in the second (Fig 6A) and, importantly, enables the quantitative assessment of species separated by 2D gel [29] (Fig 6B and 6C).

The CSF analysis (20-AD, 20-iNPH patients and 12-controls sub-pooled by combining 4 different samples randomly selected within AD, iNPH and control, as described in methods) revealed the presence of four proteoforms (see MS data in S7 Table, S2 and S3 Figs), 3 of them dysregulated (One-way ANOVA and Tukey tests, p-value<0.01) (i.e. a, pIa = 5.13; b, pIb = 5.20; c, pIc = 5.27; d, pId = 5.35; see Fig 6A, 6B and 6D). The total volume of Apo A-1 proteoforms was decreased (-29%), confirming the results of MALDI profiling.

As the total amount of Apo A-1 in serum did not change significantly, we wondered if serum of AD patients was characterized by an unbalanced proteoform distribution unaffecting the total protein amount. Five proteoforms of Apo A-1 were detected [30] (i.e. a-d, same pI as in CSF, and e, pIe = 5.42; see Fig 6A, 6B and 6C), four of them were common to CSF but the proteoform level in serum was at variance, with two proteoforms overexpressed in AD vs. iNPH and healthy controls and one proteoform decreased in iNPH, only. The proteoforms in the pI range from pH 5.13–5.27 correspond to the mature form of Apo A-1, whereas the proteoforms at pId = 5.35 and pIe = 5.42 represent the precursors of Apo A-1 (proapo) as indicated by MS analysis. The proteoforms distribution of Apo A-1 in CSF vs serum were at variance (Fig 6). In serum the proapo form e, is unchanged in AD whereas the proapo form d, is increased in AD compared to controls and iNPH together with Apo A-1 proteoform b (this proteoform was unchanged in CSF). Conversely, Apo A-1 proteoform a is unchanged in AD whereas is decreased in iNPH. In CSF, Apo A-1 proteoform a and c decreased (Fig 6B), indicating that, not only the level of total Apo A-1 but also a different distribution of Apo A-1 proteoforms characterizes serum and CSF in AD.

## Discussion

The proteomics of biological fluids is a powerful approach for the identification of altered levels of circulating proteins/peptides as a result of genetic and/or metabolic changes. Along this line, CSF followed by MALDI profiling has been utilized as preliminary screening to identify peptides or proteoforms associated to AD, to supplement the panel of already existing biomarkers to improve diagnostic sensitivity and accuracy. The proposed “top-down” proteomics allows to precisely group subjects, and to determine peaks statistically relevant to be identified. In addition, this analysis allows to process a considerable number of samples in a short time with low costs, providing simple spectra, thus reducing the burden of MS data generated by bottom-up approaches, making clinical studies affordable. The drawback of this approach is that it requires two steps: the first implies the presence of best separating peaks, and the second requires peak isolation by chromatography or gel-based fractionation and identification as described by our and other studies [23, 31, 32]. However, this approach enables a protein fingerprint of intact proteins of minute quantities of CSF. The present study shows that iNPHs share a similar CSF MALDI low molecular weight protein profile of healthy controls as no differentially expressed peaks are detected, indicating that these samples can be utilized as reference in comparative CSF analyses. Moreover, results revealed the presence of a specific protein pattern for AD patients in the m/z range 4000–35000 demonstrating that endogenous small peptides are dysregulated in CSF of AD compared to iNPH patients. Furthermore, this specific pattern is able to classify blinded samples as demonstrated by the classification model shown in Table 2 with high sensitivity and specificity.

Furthermore, we are aware that the choice of right control samples is a significant criterion for conducting a study in evidence-based medical research when case-control studies are faced. In this study iNPH were not perfectly age-matched (median age, min-max; 83.5, 70–91) with AD (median age, min-max; 77, 68–86) but, on the other hand, we considered that the comparison was performed by selecting elderly patients both for AD and iNPH.

Moreover, iNPHs did not show any statistical difference in the low molecular weight fraction even if compared to cognitively healthy controls (median age, min-max; 75.5, 55–84). Finally, the choice of iNPH as control group represented a good strategy as they are matched with AD for dementia symptoms.

In principle the best biomarker is a long half-life molecule detectable in easily collectable specimen such as serum. However, in serum, protein species associated to complex physiopathological events are often present in small concentration and remain undetectable to MS analysis unless high-abundant proteins are removed [33] (i.e. by immunodepletion). It is noteworthy that, the analysis of CSF and serum from the same patient indicated that some of the best separating peaks are present in both biological fluids with different trend (Fig 3), highlighting the relevance of our approach for prioritizing candidates for MS recognition.

Other studies, such as that of Lehallier et al. [34], after the selection and combination of existing specific demographic and clinical variables, suggested a combination of CSF and plasma biomarkers to predict the midterm progression from MCI to AD. The novelty of the proposed study relies in the application of an untargeted approach, like MALDI profiling, to screen CSF samples from controls, iNPH and AD to reveal the increment or decrement of a set of new putative protein markers characteristic of these conditions. This first step allowed to simultaneously analyse in a fast and reliable mode a number of samples which largely satisfied statistic issues.

Furthermore, results indicated a signal at 28000 m/z, identified as Apo A-1, as the “best separating peak” between CSF from ADs and iNPHs. This protein was under-expressed in AD versus iNPH and the classification model suggested that this is the essential peak for classifying AD vs iNPH. Apo A-1 was dysregulated in blinded samples utilized for the validation of the classification model (see Table 2), supporting the prominent role for this protein in AD versus iNPH.

Apo A-1 is a relatively abundant plasma protein with a concentration of 1.0–1.5 mg/mL [35], it is present in five physiological isoforms [30, 36]. Currently, the mechanisms, which cause Apo A-1 decrement in CSF of AD patients, remain unclear even if events, as alteration of membrane permeability, inefficient transport of metabolites or protein aggregation, can be hypothesized [37, 38].

Recently, a negative association with memory performance has been related to increased DNA methylation and higher serum levels of Apo A-1 [19].

At variance with CSF, serum profiling of AD patients together with total Apo A-1 quantitation by immunoblotting, revealed a non statistically significant trend in Apo A-1 level respect to iNPHs and controls, whereas the precise quantitation of proteoforms by 2-D DIGE confirmed the presence of quantitative changes of specific proteoforms in AD. A longitudinal study of quantitative differences in proteoform levels associated to the evolution from MCI toward AD is currently being undertaken in our lab.

This study highlighted the absence of statistically significant differences in CSF peptides/small proteins abundance between control and iNPH subjects in the m/z 4000–35000 range, indicating that iNPH can be utilized in comparative studies of AD, overcoming the issue of CSF withdraw.

It should be of note that, for the first time, the Apo A-1 proteoforms composition provided by 2-D DIGE revealed a specific distribution pattern in serum and CSF of AD patients. Notably, only some proteoforms were at variance in ADs versus iNPHs and controls. The Apo A-1 proteoforms b and d were increased in serum and decreased in CSF, whereas Apo A-1 proteoform c increased both in CSF and serum. Our results can be related to a recent study on the methylation of the promoter region of the APOA1 gene [19]. This epigenetic modification promotes the switch-off of the gene even though not associated with a protein decrement in serum.

In our work, the observed serum proapo proteoform decrement, together with the increment of Apo A-1 mature proteoform b, suggests a possible PTM or a polymorphism, which can lead to structurally modified proteoforms able to pass through the brain barrier. Once in the brain they can precipitate or co-aggregate with other proteins contributing to plaques formation. Studies are in progress to determine the presence of post-translational modifications and their position within protein sequence, by adopting LC- and MALDI-MS/MS techniques, to associate them to other types of dementia in order to verify the sequence coverage, and identify peptides susceptible to PTMs. Nevertheless we are aware that the small sample size and the absence of an independent replication for more confident statistics represent a major limitation of this study. More efforts from clinical units should be implemented to provide an appropriate number of samples for the validation step, which is essential to support these putative biomarkers, overcoming eventual statistical bias and the contribution of confounding effects.

## Supporting information

S1 Fig Apo A-1 identification. The spectrum of apolipoprotein A-1 was obtained after SDS-PAGE and PMF analysis of CSF.
(DOCX)

S2 Fig Apo A-1 proteoforms identification. In the upper panel the representative MS spectrum of Apolipoprotein A-1 proteoforms with the characteristic N-terminal peptide DEPPQSPWDR at m/z 1226.547 indicated with the arrow. In lower panel the MS/MS spectrum of the peak with m/z 1226.547 is shown.
(DOCX)

S3 Fig proApo A-1 proteoforms identification. Representative MS spectrum of proapolipoprotein A-1 proteoforms (upper panel). The N-terminal peptide DEPPQSPWDR, characteristic of Apo A-1 proteoforms, is absent (closeup in the lower panel).
(DOCX)

S1 Table Detailed participants’ characteristics. (PDF)

S2 Table Data used for AD CSF vs iNPH CSF comparison. Area values used by ClinProTools software to perform statistics on Apo A-1 peak in the comparison AD CSF vs iNPH CSF. The indicated values originates from normalization and smoothing operations performed by the software.
(XLSX)

S3 Table Apo A-1 identification data. Apo A-1 identification results provided by PMF approach.
(PDF)

S4 Table Data used for AD serum vs iNPH serum vs control serum comparison. Area values used by ClinProTools software to perform statistics on Apo A-1 peak in the comparison AD serum vs iNPH serum vs control serum. The indicated values originate from normalization and smoothing operations performed by the software.
(XLSX)

S5 Table 2-D DIGE volumes for Apo A-1 proteoforms in CSF. Values represent the "standardised spot abundances" calculated by DeCyder software.
(PDF)

S6 Table 2-D DIGE volumes for Apo A-1 proteoforms in serum. Values represent the "standardised spot abundances" calculated by DeCyder software.
(PDF)

S7 Table Mass spectrometry identification data for apo A-1 and proapo A-1 proteoforms. Together with proteoform name, the GI, therotichal pI and MW, method of analysis, sequence coverage, number of identified peptides and information about MS/MS analysis (where needed) are reported. In proapo proteoforms the N-terminal peptide characteristic of apo proteoforms (DEPPQSPWDR) is absent.
(PDF)

## Abbreviations

2-D DIGE: biDimensional Gel Electrophoresis
Aβ: Amyloid beta
AD: Alzheimer’s Disease
AUC: Area Under the Curve
CSF: CerebroSpinal Fluid
CV: Coefficient of Variation
iNPH: Idiophatic Normal Pressure Idrocephalus
MALDI-MS: Matrix Assisted Laser Desorption Ionization—Mass Spectrometry
MW: Molecular Weight
PCA: Principal Component Analysis
pI: Isoelectric point
PTM: Post Translational Modification
ROC: Receiver Operating Characteristic
SDS-PAGE: Sodium Dodecyl Sulphate—PolyAcrylamide Gel Electrophoresis
