# Supplementary figures and images for: Protein signature in cerebrospinal fluid and serum of Alzheimer’s disease patients: The case of apolipoprotein A-1 proteoforms
Source: PLoS One. 2017 Jun 19;12(6):e0179280. doi: 10.1371/journal.pone.0179280 (PMC5476270; doi:10.1371/journal.pone.0179280)

**Figure 1S.** PMF spectrum of apolipoprotein A-1 obtained after SDS-PAGE analysis of CSF.
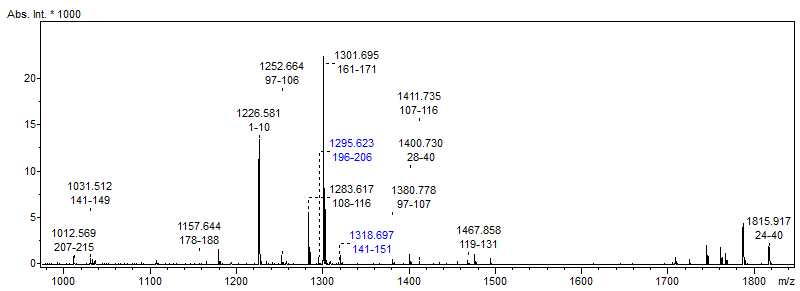

Supplement: S1 Fig — The spectrum of apolipoprotein A-1 was obtained after SDS-PAGE and PMF analysis of CSF. (DOCX) [file pone.0179280.s001.docx]
