# Supplementary material for: Protein signature in cerebrospinal fluid and serum of Alzheimer’s disease patients: The case of apolipoprotein A-1 proteoforms
Source: PLoS One. 2017 Jun 19;12(6):e0179280. doi: 10.1371/journal.pone.0179280 (PMC5476270; doi:10.1371/journal.pone.0179280)
Supplement: S2 Fig — In the upper panel the representative MS spectrum of Apolipoprotein A-1 proteoforms with the characteristic N-terminal peptide DEPPQSPWDR at m/z 1226.547 indicated with the arrow. In lower panel the MS/MS spectrum of the peak with m/z 1226.547 is shown. (DOCX) [file pone.0179280.s002.docx]

**Figure 2S.** In the upper panel the representative MS spectrum of Apolipoprotein A-1 proteoforms with the characteristic N-terminal peptide DEPPQSPWDR at m/z 1226.547 indicated with the arrow. In lower panel the MS/MS spectrum of the peak with m/z 1226.547 is shown.
