# Supplementary material for: Protein signature in cerebrospinal fluid and serum of Alzheimer’s disease patients: The case of apolipoprotein A-1 proteoforms
Source: PLoS One. 2017 Jun 19;12(6):e0179280. doi: 10.1371/journal.pone.0179280 (PMC5476270; doi:10.1371/journal.pone.0179280)
Supplement: S3 Fig — Representative MS spectrum of proapolipoprotein A-1 proteoforms (upper panel). The N-terminal peptide DEPPQSPWDR, characteristic of Apo A-1 proteoforms, is absent (closeup in the lower panel). (DOCX) [file pone.0179280.s003.docx]

**Figure 3S.** Representative MS spectrum of proapolipoprotein A-1 proteoforms (upper panel). The N-terminal peptide DEPPQSPWDR, characteristic of Apo A-1 proteoforms, is absent (closeup in the lower panel).
