# Supplementary material for: Protein signature in cerebrospinal fluid and serum of Alzheimer’s disease patients: The case of apolipoprotein A-1 proteoforms
Source: PLoS One. 2017 Jun 19;12(6):e0179280. doi: 10.1371/journal.pone.0179280 (PMC5476270; doi:10.1371/journal.pone.0179280)
Supplement: S1 Table — (PDF) [file pone.0179280.s004.pdf]

| Sample | Age (years) | MMSE | DIAGNOSIS | AB (pg/mL) | Tau (pg/mL) | p-Tau (pg/mL) |
|---|---|---|---|---|---|---|
| **1** | 86 | 17 | | 1014 | 191 | 56 |
| **2** | 70 | 25 | | 307 | 609 | 124 |
| **3** | 83 | 25 | | 372 | 1420 | 132 |
| **4** | 76 | 24 | | 391 | 999 | 136 |
| **5** | 71 | 27 | | 493 | 1316 | 87 |
| **6** | 86 | 13 | | 393 | 1266 | 70 |
| **7** | 70 | 26 | | 346 | 575 | 70 |
| **8** | 68 | 26 | | 268 | 779 | 110 |
| **9** | 76 | 27 | | 1151 | 162 | 18 |
| **10** | 76 | 21 | | 462 | 745 | 64 |
| **11** | 73 | 23 | | 454 | 206 | 22 |
| **12** | 75 | 16 | | 544 | 803 | 100 |
| **13** | 81 | 22 | | 563 | 826 | 109 |
| **14** | 70 | 26 | | 260 | 421 | 50 |
| **15** | 78 | 21,3 | | 226 | 423 | 80 |
| **16** | 75 | 16 | | 590 | 721 | 65 |
| **17** | 81 | 24 | | 414 | 419 | 68 |
| **18** | 80 | 19 | AD | 322 | 208 | 28 |
| **19** | 82 | 26 | | 354 | 567 | 81 |
| **20** | 71 | 18 | | 748 | 578 | 59 |
| **21** | 81 | 25 | | 499 | 153 | 45 |
| **22** | 78 | 25 | | 516 | 110 | 33 |
| **23** | 79 | 22 | | 532 | 276 | 73 |
| **24** | 75 | 27 | | 546 | 491 | 101 |
| **25** | 75 | 21 | | 492 | 163 | 41 |
| **26** | 80 | 24 | | 654 | 646 | 78 |
| **27** | 79 | 23 | | 504 | 572 | 52 |
| **28** | 82 | 22 | | 447 | 146 | 77 |
| **29** | 82 | 25 | | 509 | 512 | 75 |
| **30** | 68 | 13 | | 472 | 308 | 68 |
| **31** | 73 | 18 | | 275 | 2952 | 475 |
| **32** | 80 | 24 | | 420 | 526 | 66 |
| **33** | 70 | 26 | | 449 | 1524 | 155 |
| **34** | 78 | 25 | | 288 | 1023 | 66 |
| **35** | 74 | 28 | | 627 | 2126 | 155 |
| **36** | 78 | 29 | | 624 | 842 | 171 |
| **37** | 90 | 23 | | 453 | 909 | 102 |
| **38** | 87 | 20 | | 456 | 934 | 128 |
| **39** | 84 | 17 | | 563 | 826 | 109 |
| **40** | 85 | 13 | | 260 | 421 | 50 |
| **41** | 84 | 24 | | 861 | 501 | 19 |
| **42** | 77 | 24 | | 859 | 284 | 42 |
| **43** | 85 | 18 | | 997 | 496 | 37 |
| **44** | 81 | 24 | | 900 | 256 | 45 |
| **45** | 83 | 29 | | 1248 | 125 | 26 |
| **46** | 90 | 22 | | 371 | 600 | 59 |
| **47** | 75 | 23 | | 800 | 100 | 20 |
| **48** | 88 | 24 | | 790 | 157 | 28 |

| | | | | | | |
|---|---|---|---|---|---|---|
| **49** | 91 | 30 | iNPH | 586 | 133 | 15 |
| **50** | 86 | 26 | | 225 | 422 | 80 |
| **51** | 84 | 24 | | 590 | 721 | 65 |
| **52** | 82 | 27 | | 450 | 682 | 58 |
| **53** | 83 | 28 | | 1158 | 99 | 15 |
| **54** | 87 | 21 | | 661 | 676 | 73 |
| **55** | 85 | 29 | | 610 | 75 | 15 |
| **56** | 70 | 23 | | 1010 | 130 | 15 |
| **57** | 90 | 25 | | 808 | 98 | 17 |
| **58** | 75 | 25 | | 250 | 252 | 52 |
| **59** | 83 | 27 | | 1120 | 159 | 29 |
| **60** | 73 | 23 | | 526 | 46 | 24 |
| **61** | 79 | 22 | | 1061 | 74 | 24 |
| **62** | 84 | 24 | | 946 | 76 | 29 |
| **63** | 86 | 24 | | 1570 | 146 | 51 |
| **64** | 81 | 28 | | 819 | 115 | 35 |
| **65** | 85 | 29 | | 614 | 67 | 25 |
| **66** | 86 | 15 | | 747 | 102 | 29 |
| **67** | 80 | 27 | | 418 | 250 | 26 |
| **68** | 83 | 23 | | 226 | 423 | 79 |
| **LC1** | 77 | - | CT | 453 | 49 | 10 |
| **LC2** | 73 | - | | 581 | 54 | 7 |
| **LC3** | 78 | - | | 457 | 111 | 13 |
| **LC4** | 77 | - | | 853 | 85 | 14 |
| **LC5** | 80 | - | | 965 | 96 | 25 |
| **LC6** | 84 | - | | 1515 | 115 | 30 |
| **LC7** | 74 | - | | 1306 | 103 | 26 |
| **LC8** | 70 | - | | 1001 | 158 | 40 |
| **LC10** | 68 | - | | 1424 | 95 | 31 |
| **LC11** | 82 | - | | 1300 | 149 | 37 |
| **LC12** | 55 | - | | 882 | 75 | 29 |
| **LC13** | 69 | - | | 1062 | 94 | 35 |

**S1 Table. Detailed participants' characteristics.**
