# Supplementary material for: Protein signature in cerebrospinal fluid and serum of Alzheimer’s disease patients: The case of apolipoprotein A-1 proteoforms
Source: PLoS One. 2017 Jun 19;12(6):e0179280. doi: 10.1371/journal.pone.0179280 (PMC5476270; doi:10.1371/journal.pone.0179280)
Supplement: S3 Table — Apo A-1 identification results provided by PMF approach. (PDF) [file pone.0179280.s006.pdf]

Protein name	MW [kDa]	pI	Searched/ Matched peptides	Mascot score	Sequence coverage	m/z measured	Mr calculated	z	Δ m/z [ppm]	Sequence
						1012.5530	1011.5713	1	-25.29	**231**AKPALEDLR**239**
						1157.6099	1156.6200	1	-15.00	**202**LEALKENGGAR**212**
						1252.5954	1251.6136	1	-20.33	**121**VQPYLDDFQK**130**
						1283.5491	1282.5652	1	-18.19	**132**WQEEMELYR**140**
						1301.6250	1300.6412	1	-18.03	**185**THLAPYSDELR**195**
Apolipoprotein A-1	30.8	5.5	11/28	121	34.5	1318.6207	1317.6347	1	-16.11	**165**LSPLGEEMRDR**175**
						1380.6914	1379.7085	1	-17.70	**121**VQPYLDDFQKK**131**
						1400.6567	1399.6620	1	-8.97	**52**DYVSQFEGSALGK**64**
						1411.6502	1410.6602	1	-12.24	**131**KWQEEMELYR**140**
						1467.7740	1466.7841	1	-11.83	**143**VEPLRAELQEGAR**155**
						1815.8479	1814.8435	1	-1.58	**48**DSGRDYVSQFEGSALGK**64**

S3 Table. Apo A-1 identification data. Apo A-1 identification results provided by PMF approach.
