# Supplementary material for: Protein signature in cerebrospinal fluid and serum of Alzheimer’s disease patients: The case of apolipoprotein A-1 proteoforms
Source: PLoS One. 2017 Jun 19;12(6):e0179280. doi: 10.1371/journal.pone.0179280 (PMC5476270; doi:10.1371/journal.pone.0179280)
Supplement: S5 Table — Values represent the "standardised spot abundances" calculated by DeCyder software. (PDF) [file pone.0179280.s008.pdf]

S5 Table. 2-D DIGE volumes for Apo A-1 proteoforms in CSF. Values represent the "standardised spot abundances" calculated by DeCyder software.

		AD					iNPH					CT		
**Proteoform**	**Technical replicate**	**subpool 1**	**subpool 2**	**subpool 3**	**subpool 4**	**subpool 5**	**subpool 1**	**subpool 2**	**subpool 3**	**subpool 4**	**subpool 5**	**subpool 1**	**subpool 2**	**subpool 3**
**a**	1	0,812300	0,951192	0,908985	0,944458	0,901331	1,222856	1,218046	1,252496	1,220299	1,258486	3,371422	3,405803	2,910112
	2	0,932300	0,940897	0,908798	0,914051	0,892496	1,252159	1,199865	1,247482	1,210269	1,247221	3,363633	3,392919	2,900180
	3	0,870500	0,957292	0,915199	0,919917	0,889592	1,240479	1,209839	1,245693	1,203173	1,252856	3,353426	3,399811	2,896226
**b**	1	0,881616	0,819112	0,849019	0,847519	0,869187	1,289186	1,374682	1,257546	1,362826	1,230525	1,161453	1,290413	1,230048
	2	0,890989	0,835187	0,837997	0,822579	0,867542	1,278859	1,356844	1,264063	1,365222	1,244740	1,174544	1,287498	1,218045
	3	0,899647	0,830744	0,834721	0,832415	0,881856	1,279856	1,361101	1,259218	1,373460	1,238522	1,167281	1,279932	1,212059
**c**	1	0,799548	0,850127	0,830275	0,830278	0,841294	1,199858	1,240611	1,216534	1,244135	1,201456	0,366411	0,411256	0,346791
	2	0,810997	0,842094	0,819894	0,806496	0,831286	1,215049	1,232503	1,206246	1,241019	1,193746	0,379414	0,417024	0,351127
	3	0,812521	0,852214	0,823528	0,809697	0,842885	1,209596	1,224215	1,208476	1,233218	1,188445	0,374795	0,420114	0,358956
**d**	1	0,935647	0,871243	0,832475	0,880054	0,882146	1,129213	1,160429	1,181268	1,114452	1,179846	1,721596	1,703446	1,687440
	2	0,929985	0,861224	0,821812	0,872037	0,873144	1,119945	1,154622	1,189486	1,120046	1,191293	1,696912	1,691426	1,678435
	3	0,941054	0,875225	0,827294	0,878941	0,877649	1,123671	1,174116	1,192042	1,126173	1,185580	1,701883	1,695248	1,674126

**average volume proteoform a**	0,871700	0,949794	0,910994	0,926142	0,894473	1,238498	1,209250	1,248557	1,211247	1,252854	3,362827	3,399511	2,902173
**average volume proteoform b**	0,890751	0,828348	0,840579	0,834171	0,872862	1,282634	1,364209	1,260276	1,367169	1,237929	1,167759	1,285948	1,220051
**average volume proteoform c**	0,807689	0,848145	0,824566	0,815490	0,838488	1,208168	1,232443	1,210419	1,239457	1,194549	0,373540	0,416131	0,352291
**average volume proteoform d**	0,935562	0,869231	0,827194	0,877011	0,877646	1,124276	1,163056	1,187599	1,120224	1,185573	1,706797	1,696707	1,680000

	**Average AD**	**St. dev. AD**	**Average iNPH**	**St. dev. iNPH**	**Average CT**	**St. dev. CT**
**proteoform a**	0,910621	0,029796	1,232081	0,020612	3,22150	0,27716
**proteoform b**	0,853342	0,027090	1,302443	0,059869	1,22459	0,05922
**proteoform c**	0,826876	0,016510	1,217007	0,018495	0,38065	0,03251
**proteoform d**	0,877329	0,038634	1,156145	0,032439	1,69450	0,01353
