# Supplementary material for: Protein signature in cerebrospinal fluid and serum of Alzheimer’s disease patients: The case of apolipoprotein A-1 proteoforms
Source: PLoS One. 2017 Jun 19;12(6):e0179280. doi: 10.1371/journal.pone.0179280 (PMC5476270; doi:10.1371/journal.pone.0179280)
Supplement: S6 Table — Values represent the "standardised spot abundances" calculated by DeCyder software. (PDF) [file pone.0179280.s009.pdf]

S6 Table. 2-D DIGE volumes for Apo A-1 proteoforms in Serum. Values represent the "standardised spot abundances" calculated by DeCyder software.

		AD					iNPH					CT		
**Proteoform**	**Technical replicate**	**subpool 1**	**subpool 2**	**subpool 3**	**subpool 4**	**subpool 5**	**subpool 1**	**subpool 2**	**subpool 3**	**subpool 4**	**subpool 5**	**subpool 1**	**subpool 2**	**subpool 3**
**a**	1	1,041996	1,151453	1,134575	1,015879	1,172456	0,953696	0,980125	1,070024	1,154256	0,896262	1,011243	1,058766	1,180126
	2	1,058988	1,221608	0,974586	0,900756	1,301453	0,889453	0,866453	1,009807	1,030486	0,961457	1,066192	0,929845	1,053452
	3	1,144862	1,284265	1,053821	0,957288	1,239926	1,024533	0,924199	0,947124	1,092673	0,842843	1,134586	0,988456	1,118106
**b**	1	1,138465	1,274523	1,157194	1,248846	1,051246	0,940468	0,972798	0,913361	0,952865	0,930622	1,050835	1,043802	1,061088
	2	1,079072	1,138453	1,227456	1,182098	1,182456	1,000549	1,036453	0,975126	0,890453	0,993452	0,989922	1,104812	0,998453
	3	1,049146	1,190213	1,097453	1,118753	1,125761	0,880045	0,910457	0,847653	1,012446	0,871824	1,114596	0,969803	1,125466
**c**	1	0,974257	1,158393	1,152781	1,169453	1,099038	0,826338	0,885421	0,804236	0,769843	0,810011	1,054695	1,101453	1,039846
	2	1,100787	1,228456	1,200144	1,031453	1,018453	0,884528	0,819695	0,860144	0,885453	0,746255	0,921447	0,995486	1,114541
	3	1,032856	1,089845	1,101453	1,100591	1,151243	0,768453	0,757864	0,748566	0,826345	0,874543	0,99229	1,051478	0,971581
**d**	1	1,021488	1,222747	1,178453	1,180185	1,271453	0,982433	0,950751	0,975435	1,045455	0,955736	0,944672	1,128846	1,010456
	2	1,172481	1,305426	1,310456	1,249843	1,215531	0,866459	1,015426	0,915363	0,915469	0,891312	0,872454	0,999053	1,120453
	3	1,131798	1,165426	1,248012	1,111456	1,142489	0,925293	0,885429	0,857612	0,982211	0,825476	0,995469	1,061736	1,065411
**e**	1	0,864113	0,975734	1,021459	0,928453	0,915813	0,936154	1,201455	1,145783	1,256541	0,949266	0,986118	0,851841	0,994318
	2	0,939115	0,897435	0,962239	0,965974	0,974563	0,998476	1,311476	1,269452	1,320451	0,884525	0,912488	0,791442	1,054765
	3	1,012311	1,051453	0,895426	1,084256	0,839453	0,872498	1,259173	1,208161	1,189422	1,012455	1,035496	0,912453	0,929453

**average volume proteoform a**	1,081949	1,219109	1,054327	0,957974	1,237945	0,955894	0,923592	1,008985	1,092472	0,900187	1,070674	0,992356	1,117228
**average volume proteoform b**	1,088894	1,201063	1,160701	1,183232	1,119821	0,940354	0,973236	0,912047	0,951921	0,931966	1,051784	1,039472	1,061669
**average volume proteoform c**	1,035967	1,158898	1,151459	1,100499	1,089578	0,826440	0,820993	0,804315	0,827214	0,810270	0,989477	1,049472	1,041989
**average volume proteoform d**	1,108589	1,231200	1,245640	1,180495	1,209824	0,924728	0,950535	0,916137	0,981045	0,890841	0,937532	1,063212	1,065440
**average volume proteoform e**	0,938513	0,974874	0,959708	0,992894	0,909943	0,935709	1,257368	1,207799	1,255471	0,948749	0,978034	0,851912	0,992845

	**Average AD**	**St. dev. AD**	**Average iNPH**	**St. dev. iNPH**	**Average CT**	**St. dev. CT**
**proteoform a**	1,110261	0,117550	0,976226	0,076733	1,06009	0,06311
**proteoform b**	1,150742	0,046012	0,941905	0,022774	1,05098	0,01112
**proteoform c**	1,107280	0,050153	0,817846	0,010149	1,02698	0,03269
**proteoform d**	1,195150	0,054249	0,932657	0,034443	1,02206	0,07321
**proteoform e**	0,955186	0,032229	1,121019	0,164481	0,94093	0,07745
