# Supplementary material for: Protein signature in cerebrospinal fluid and serum of Alzheimer’s disease patients: The case of apolipoprotein A-1 proteoforms
Source: PLoS One. 2017 Jun 19;12(6):e0179280. doi: 10.1371/journal.pone.0179280 (PMC5476270; doi:10.1371/journal.pone.0179280)
Supplement: S7 Table — Together with proteoform name, the GI, therotichal pI and MW, method of analysis, sequence coverage, number of identified peptides and information about MS/MS analysis (where needed) are reported. In proapo proteoforms the N-terminal peptide characteristic of apo proteoforms (DEPPQSPWDR) is absent. (PDF) [file pone.0179280.s010.pdf]

S7 Table. Mass spectrometry identification data for apo A-1 and proapo A-1 proteoforms. Together with proteoform name, the GI, therotichal pI and MW, method of analysis, sequence coverage, number of identified peptides and information about MS/MS analysis (where needed) are reported. In proapo proteoforms the N-terminal peptide characteristic of apo proteoforms (DEPPQSPWDR) is absent.

Proteoform	Name	GI	Theroetichal MW [kDa]	Theorethical pI	Method	MASCOT score	Sequence Coverage (%)	Matched/ searched peptides	MS/MS	MS/MS Score	m/z	z
a	Apolipoprotein A-1	90108664	28.1	5.1	PMF+ MS/MS	102	47	10/19	**1**DEPPQSPWDR**10**	55	1226.544	1
b						205	49	14/23		56	1226.538	1
c						276	71	22/38		72	1226.544	1
d	Proapolipoprotein A-1	253362	28.9	5.45	PMF	172	65	21/38	**absent**			
e						175	36	11/16				
